# Low rate of rescue epidural analgesia after open colorectal surgery with intrathecal morphine: a retrospective cohort study

**DOI:** 10.1007/s00384-025-04833-w

**Published:** 2025-02-13

**Authors:** Sebastian de Brun, Abbas Chabok, Malin Engdahl, Erland Östberg

**Affiliations:** 1 Department of Anaesthesia and Intensive Care, Västmanland Hospital Västerås, 721 89 Västerås, Sweden; 2 Region Västmanland – Uppsala University, Centre for Clinical Research, Västmanland Hospital Västerås, Västerås, Sweden; 3 Division of Surgery, Danderyd University Hospital, Stockholm, Sweden; 4 Department of Surgery, Västmanland Hospital Västerås, Västerås, Sweden

**Keywords:** Injections, Spinal, Morphine, Laparotomy, Colorectal surgery

## Abstract

**Purpose:**

The use of intrathecal morphine in open colorectal surgery has been limited despite being a promising analgesic alternative used in other types of open abdominal surgery. Intrathecal morphine has a higher success rate than thoracic epidural analgesia, the current standard method of analgesia in open colorectal surgery. Intrathecal morphine is occasionally used in open colorectal surgery when thoracic epidural analgesia placement fails and in instances when patients receive intrathecal morphine for a planned laparoscopic surgical procedure which is converted to laparotomy intraoperatively. This retrospective single-centre cohort study aimed to evaluate outcomes after intrathecal morphine in patients undergoing open colorectal surgery.

**Methods:**

All patients who received intrathecal morphine before open colorectal surgery at a secondary hospital in Sweden between 2016 and 2020 were included. Routinely collected data from the Swedish PeriOperative Registry and patients’ medical records were reviewed, and data regarding postoperative outcomes including the incidence of postoperative rescue thoracic epidural analgesia and adverse events were extracted.

**Results:**

In total, 108 patients were included with a median age of 74 years. Four patients (4%) received rescue thoracic epidural analgesia postoperatively, and the median hospital length of stay was 8 days. The median intrathecal morphine dose was 200 µg. Respiratory complications occurred in two patients (2%).

**Conclusion:**

The incidence of rescue thoracic epidural analgesia after intrathecal morphine in open colorectal surgery was low, and there were few adverse events. The results suggest that intrathecal morphine could be a viable alternative for postoperative pain management in open colorectal surgery.

**Supplementary Information:**

The online version contains supplementary material available at 10.1007/s00384-025-04833-w.

## Introduction

Surgery is the mainstay treatment for colorectal cancer and is also a common treatment for inflammatory bowel disease and diverticulitis. Even though the frequency of laparoscopic and robot-assisted procedures is increasing, a considerable part of colorectal surgical procedures are still performed open [1]. The introduction of enhanced recovery protocols has facilitated recovery after colorectal surgery by reducing stress and maintaining physiological functions [2]. Adequate postoperative analgesia is an important part of these protocols as postoperative pain is associated with impaired recovery, increased morbidity and reduced quality of life [3, 4]. The Enhanced Recovery After Surgery (ERAS®) Society recommends thoracic epidural analgesia (TEA) as standard management of postoperative pain in open colorectal surgery [5]. However, the modestly improved pain reduction of TEA in comparison to intravenous analgesia may be outweighed by high failure rates [6–9].

Intrathecal hydrophilic opioids such as morphine are an effective and commonly used method of analgesia in both open and laparoscopic abdominal surgery [10, 11] Intrathecal morphine (ITM) shows a similar reduction in pain as TEA in open abdominal hysterectomy and liver resection [12–14]. On the other hand, studies comparing ITM with TEA in open colorectal surgery are rare and the limited evidence requires retrospective mapping of current use before designing a prospective trial.

This retrospective observational study aimed to evaluate outcomes for patients who received ITM preoperatively and underwent open colorectal surgery. The hypothesis was that the incidence of postoperative rescue TEA is low and adverse events are few.

## Methods

This was a retrospective cohort study evaluating routinely collected data that were registered in the Swedish PeriOperative Registry and in patients’ medical records at Västmanland Hospital Västerås. This study was reported according to the Strengthening the Reporting of Observational Studies in Epidemiology (STROBE) statement [15].

Västmanland Hospital Västerås is a public secondary hospital with a catchment area of 280,000 residents and serves the county of Västmanland for all colorectal surgery. Approximately 380 colorectal procedures are performed each year in both acute and elective settings. Colorectal procedures commonly performed at the hospital include resective bowel surgery such as colectomy, anterior resection and abdominoperineal rectum excision. The procedures are performed as open or laparoscopic surgery, including robot-assisted surgery. Following departmental protocol, preoperatively placed TEA is the method of choice for pain management in open colorectal surgery. However, ITM is occasionally used in both acute and elective open colorectal surgery for patients with relative contraindications for TEA, when TEA placement fails, and in patients planned for laparoscopic colorectal surgery that is converted to laparotomy intraoperatively. Regardless of the chosen preoperative or perioperative analgesia technique, postoperative TEA is our primary rescue approach for managing moderate to severe pain following laparotomy. If a neuraxial blockade is contraindicated or cannot be successfully placed, we instead use patient-controlled analgesia (PCA) with intravenous morphine and/or administer a peripheral nerve block as needed.

All patients aged 18 years and older who underwent open colorectal surgery between April 2016 and June 2020 were screened for eligibility. The study start date was set to the time when ITM was first introduced in the department. Thus, during the study period, all patients who received preoperative ITM followed by planned open surgery or laparoscopic surgery converted to open surgery were included in the study. Patients who received combined ITM and TEA preoperatively were excluded. Open colorectal surgery was defined as laparotomy with a midline incision.

The records of all included patients were surveyed. Using a predefined protocol, patient data and procedure-related information were extracted. Baseline data collection included age, sex, body mass index, American Society of Anesthesiologists physical status classification, comorbidities and medications. The indication for using ITM was registered as well as the administered dose and any adjuvants. Furthermore, information was collected regarding the primary method of anaesthesia, vasopressor usage, type of additional analgesics, surgery priority, indications for surgery, type of incision, type of surgery, duration of surgery and whether the operation was converted from laparoscopic to open surgery. In addition, data regarding postoperative anaesthesiologic and surgical complications were registered.

The primary outcome was the incidence of postoperative rescue TEA. Secondary outcomes included postoperative pain scores using 11-point Numeric Rating Scale (NRS), hospital length of stay (LoS), post-dural puncture headache (PDPH), postoperative nausea and vomiting (PONV), intensive care unit (ICU) stay, surgical complications using the Clavien-Dindo classification and respiratory depression defined as peripheral oxygen saturation (SpO_2_) < 90%, respiratory frequency < 8 or treatment with naloxone due to respiratory insufficiency [16]. Follow-up time was 30 days postoperatively. Any patient undergoing a second procedure after the follow-up time and meeting the inclusion criteria for the study was included as a new patient.

### Statistical analysis

Descriptive statistics were used. Categorical variables were reported as numbers and percentages, and continuous variables were reported as medians and ranges.

## Results

### Study population

In total, 1589 operations were assessed for eligibility. Of these, 111 patients fulfilled the inclusion criteria, whereas three patients were excluded because they were planned beforehand to receive TEA postoperatively. The remaining 108 patients were included in the final analysis. Median age was 74 years (range 26–92) and 57 (53%) patients were female. Patient characteristics including comorbidities and use of prescription pain medicines are outlined in Table 1.

**Table 1:** Patient characteristics

	*n* = 108
Age, years, median (range)	74 (26–92)
Sex, *n* (%)	
Female	57 (53)
Male	51 (47)
Body mass index, kg/m^2^, median (range)	26 (13–45)
ASA^a^ classification, *n* (%)	
Class 1	5 (5)
Class 2	58 (53)
Class 3	40 (37)
Class 4	5 (5)
Smokers, *n* (%)	15 (14)
Comorbidities, *n* (%)	
Hypertension	66 (61)
Ischaemic heart disease	13 (12)
Heart failure	5 (5)
Peripheral vascular disease	1 (1)
Kidney failure	20 (18)
Diabetes mellitus	16 (15)
Chronic obstructive pulmonary disease	7 (6)
Chronic pain	12 (11)
Previous abdominal surgery	67 (62)
Current use of pain medication^b^, *n* (%)	23 (21)

^a^American Society of Anesthesiologists Physical Status. ^b^Pain medications including but not limited to paracetamol, nonsteroidal anti-inflammatory drugs, opioids, gabapentinoids, alpha-2 adrenergic agonists and tricyclic antidepressants

### Surgical interventions

Surgery was performed electively in 79 patients (73%) and acute in 29 patients (27%). Median surgery time was 219 min (range 40–520 min). In 56 patients (52%), surgery was converted from laparoscopic technique to laparotomy. Colorectal resection was performed in 92 patients (85%), with primary anastomosis in 58 and stoma in 34 patients. Sixteen patients (15%) underwent laparotomy without resection of whom 11 received a stoma. The indications for surgery were malignancy in 73 patients (68%), diverticular disease including fistulas in 13 patients (12%), inflammatory bowel disease in eight patients (7%) and other diagnoses in 14 patients (13%).

### Intrathecal morphine

Indications for ITM were planned laparoscopic surgery in 56 patients (52%), failed TEA placement in 10 patients (9%), uncertain haemostasis in two patients (2%) and not specified in 42 patients (37%). Of the 42 patients without a specified indication for ITM, 23 patients (55%) underwent emergency surgery. The ITM dose was 200 µg in 93 patients (86%), between 100 and 200 µg in eight patients (8%) and not documented in seven (6%) patients.

Intrathecal adjuvants were bupivacaine in 86 patients (79%), median dose 4.5 mg (range 1–10 mg); clonidine in 10 patients (9%), median dose 75 µg (range 45–150 µg); fentanyl 10 µg in five patients (5%); and ropivacaine 5 mg in one patient (1%).

### Anaesthesia and additional analgesics

General anaesthesia was maintained with inhalational agents in 57 patients (53%), and total intravenous anaesthesia was used in 51 patients (47%). Vasopressors were used in 107 patients (99%). Additional analgesics used as part of multimodal analgesia are outlined in Table 2. One patient with ITM received an additional transversus abdominis plane block after the decision on conversion from laparoscopic to open surgery. No patients received postoperative peripheral nerve blocks or PCA with intravenous morphine.

**Table 2:** Additional analgesics administered

	*n* = 108
Preoperative oral or intravenous analgesics, *n* (%)	
Gabapentin	17 (16)
Opioids^a^	3 (3)
Paracetamol	5 (5)
Steroids	3 (3)
Intraoperative intravenous analgesics, *n* (%)	
Clonidine	5 (5)
Esketamine^b^	13 (12)
Lidocaine^b^	2 (2)
NSAIDs^c^	7 (6)
Opioids^d^	104 (96)
Paracetamol	55 (51)
Steroids	35 (32)
Postoperative oral or intravenous analgesics, *n* (%)	
Clonidine	6 (6)
Esketamine^b^	6 (6)
Gabapentin	23 (22)
Lidocaine^b^	2 (2)
NSAIDs^c^	2 (2)
Opioids^e^	108 (100)
Paracetamol	107 (99)
Steroids	5 (5)

^a^Morphine and/or oxycodone; ^b^administered as bolus and/or infusion; ^c^nonsteroidal anti-inflammatory drugs; ^d^remifentanil, alfentanil, fentanyl and/or morphine; ^e^morphine, ketobemidone and/or oxycodone

### Outcomes

Of the 108 patients who received ITM preoperatively and underwent open colorectal surgery, four patients (4%) received postoperative rescue TEA because of inadequate analgesia. The remaining 104 patients (96%) received only oral or intravenous analgesics as part of multimodal analgesia. Records of postoperative pain scores using NRS were available for 43 patients. At arrival in PACU, the median NRS (IQR) was 0 (0–0) and at discharge from PACU 0 (0–1). The highest pain score during the PACU stay was median NRS (IQR) 3 (0–4). Respiratory depression occurred in two patients. One patient was treated with oxygen for desaturation in the postoperative care unit. The other patient was treated with naloxone and oxygen for apnoea and desaturation in close temporal relation to receiving intravenous opioids on the ward on postoperative day 2. No patients were reported to suffer from PDPH. Three patients stayed in the ICU due to reasons unrelated to ITM. PONV was registered for nine patients (8%). Postoperative surgical complications in grades three and four according to the Clavien-Dindo grading system were registered in nine patients (8%) and two patients (2%) respectively; no grade five complications were registered. Median LoS was 8 days (range 3–34 days).

## Discussion

The main findings of this retrospective cohort study of patients receiving preoperative ITM in open colorectal surgery were the low incidence of postoperative rescue TEA and few adverse events. For most patients with recorded postoperative pain scores, the values were low, indicating only mild pain. The findings indicate that ITM most often produces sufficient pain relief and is a safe method that could be a viable alternative to TEA for postoperative pain management in open colorectal surgery.

The failure rate of 4% for ITM in our study of open colorectal surgery is comparable to the failure rate of 3% for ITM in open hysterectomy [13]. TEA has higher failure rates of 7–32% reported in the literature [6–9]. When compared to TEA, ITM is an effective method of analgesia in open abdominal surgery for hysterectomy and hepatic procedures [12–14]. However, ITM was found to be inferior to TEA in open gastrectomy [17]. Open colorectal surgeries are commonly performed with a large midline incision above and below the umbilicus, while hysterectomy, gastrectomy and hepatic procedures are commonly performed with a smaller midline incision and/or a transverse incision. The different types of incisions and the different visceral parts involved may influence pain experience and make it difficult to generalize results from other studies to the population undergoing open colorectal surgery. The lack of evidence regarding ITM in open colorectal surgery warranted this study before designing a prospective trial. To date, there is no published randomized controlled trial comparing ITM and TEA in open colorectal surgery.

The mean LoS of 8 days in our study population is shorter than the 11-day LoS for the subgroup receiving ITM before open colorectal surgery reported by a previous retrospective study [18]. In subgroups that received TEA before open colon surgery and open rectal surgery in an elective setting, shorter mean LoS of 5 and 7 days respectively have been reported [7]. It is important to note that a valid comparison of these results is difficult to evaluate because a considerable part of our study population underwent emergency surgery.

Two cases of respiratory depression were detected and managed according to guidelines without negative outcomes. Although there was a temporal association with intravenous morphine administration, we cannot exclude the possibility that ITM contributed. The incidence of respiratory depression has been reported to be 0.5% for low dose (< 500 µg) ITM in abdominal surgery, and the incidence of respiratory depression has been reported to be 1.2% for TEA in abdominal surgery [6, 10]. However, comparing these results is challenging due to the small sample size in our study and the varying definitions of respiratory depression across different studies. Regarding other potential side effects, no cases of PDPH were registered and the incidence of PONV at 8% was lower than the incidence of 34% that has been reported for TEA in abdominal surgery [6].

The most common dose of ITM in our study was 200 µg which is within the dose range for most studies of ITM in abdominal surgery, although there is a lack of evidence regarding optimal dose for open colorectal procedures [10]. The wide range of adjuvants in different doses used in this study reflects the lack of evidence-based guidelines for ITM and any adjuvants. The use of additional analgesics in this population and in our practice varies, although most patients received paracetamol and opioids.

Study limitations include the retrospective design which limits the analysis to available data that are strongly dependent on what clinicians and nursing staff have documented. Examples of incompletely documented information include patients’ pain ratings and postoperative quantity of opioids administered; consequently, this information cannot be reported in full. In addition, some patients may have indicated the need for rescue TEA, but the anaesthesiologist may have opted not to, due to failed preoperative TEA placement or uncertain haemostasis. However, most patients in this study received ITM due to planned laparoscopic surgery and likely would have received a postoperative rescue TEA if an indication had occurred. Further limitations include missing information regarding the indication for ITM in several patients, as well as non-standardized dosing of ITM, intrathecal adjuvants and systemic analgesia. However, despite a suboptimal systemic analgesia regimen with a low rate of nonsteroidal anti-inflammatory drugs (NSAIDs) administered, the findings of low pain scores and low rate of rescue TEA support the hypothesis that ITM provides sufficient analgesia for most patients undergoing open colorectal surgery.

Because of the small number of cases that received postoperative rescue TEA, statistical analyses searching for predictive variables for certain outcomes were not suitable. Another limitation is that we did not include a control group with TEA, which could have made a comparison possible between ITM and TEA regarding the incidence of analgesic method failure, complications and side effects. The strength of this study is the inclusion of every patient who received preoperative ITM and underwent open colorectal surgery since its first introduction to our hospital in 2016.

This study contributes a description of how ITM has been used in open colorectal surgery and can be of value when designing future prospective studies.

## Conclusions

This retrospective cohort study evaluating the use of ITM in open colorectal surgery found a low incidence of postoperative rescue TEA and few adverse events. The results suggest that ITM could be a viable alternative to TEA for postoperative pain management in open colorectal surgery. Prospective randomized trials are needed to confirm this conclusion.

## Supplementary Information

Below is the link to the electronic supplementary material.

Supplementary file1 (DOCX 34 KB)

## Data Availability

No datasets were generated or analysed during the current study.
